# Cognitive Plasticity in Foraging *Vespula germanica* Wasps

**DOI:** 10.1673/031.011.10301

**Published:** 2011-08-18

**Authors:** Paola D'Adamo, Mariana Lozada

**Affiliations:** Laboratory Ecotono-INIBIOMA, Quintral 1250-(8400), Bariloche, Argentina

**Keywords:** learning, social wasps, visual patterns

## Abstract

*Vespula germanica* (F.) (Hymenoptera: Vespidae) is a highly invasive social wasp that exhibits a rich behavioral repertoire in which learning and memory play a fundamental role in foraging. The learning abilities of these wasps were analyzed while relocating a food source and whether *V. germanica* foragers are capable of discriminating between different orientation patterns and generalizing their choice to a new pattern. Foraging wasps were trained to associate two different stripe orientation patterns with their respective food locations. Their response to a novel configuration that maintained the orientation of one of the learned patterns but differed in other aspects (e.g. width of stripes) was then evaluated. The results support the hypothesis that *V. germanica* wasps are able to associate a particular oriented pattern with the location of a feeder and to generalize their choice to a new pattern, which differed in quality, but presented the same orientation.

## Introduction

*Vespula germanica* (F.) (Hymenoptera: Vespidae) is a highly invasive social wasp that has successfully colonized diverse environments around the world (Archer 1998). Since 1980, when it was first observed in Northwestern Patagonia, Argentina, it has spread widely throughout this region (D'Adamo et al. 2002). It has been suggested that features promoting invasion frequently entail phenotypic plasticity, since invaders have to deal with a variety of environmental conditions (Sakai et al. 2001). Invasive species, in general, repeatedly encounter novel and varying contexts for which plastic cognitive systems may be useful. These changing environments allow the association of diverse sensory-motor patterns with specific contextual traits, where “context” refers to a set of cues (physical, temporal, and motivational characteristics) in the area where a task is carried out (Cheng 2005).

Social insects exhibit great variability in foraging behavior strategies, which include flexible cognitive abilities (e.g. Raveret-Richter 2000; Giurfa et al. 2001; Goubault et al. 2005; Giurfa 2007; Menzel 2009). When exploiting a food source, foraging hymenopterans learn cues from the environment in order to retrieve memories related to rewarding stimuli (Cheng et al. 1986; Collett and Zeil 1998; Cheng 2000; Collet et al. 2003). *Vespula germanica*, in particular, is a social wasp that exhibits diverse foraging strategies as it preys on live insects, scavenges on carrion, and feeds from large stationary sources such as fruit, flowers, and honeydew (Greene 1991). As part of its social life, forager wasps collect food and take it to the nest to feed larvae. Therefore, locating a previously discovered food source and making several trips from the nest to the food location is a frequent behavior pattern in a forager's life. Being able to return to a constant place for food implies important learning and memory capacities as insects have to memorize characteristics of the food source, the route to the goal, and the specific spatial location of the food source with respect to local landmarks. Relocation behavior has been studied in *V. germanica*, providing evidence of diverse cognitive mechanisms related to this foraging strategy (D'Adamo and Lozada 2003, 2007; Lozada and D'Adamo 2006, 2009). Indeed, *V. germanica* wasps display different learning abilities when feeding in open or closed habitats (D'Adamo and Lozada 2007) as well as when exploiting protein or carbohydrate resources (D'Adamo and Lozada 2003, 2008). Moreover, they rapidly extinguish learned contexts that are no longer rewarding (Lozada and D'Adamo 2006) and show preference for the most recent positively reinforced cues (Lozada and D'Adamo 2009). In addition, these wasps integrate old and new experiences after very few learning episodes (D'Adamo and Lozada 2009). These various cognitive mechanisms could be related to the great variety of ecological environments they inhabit throughout North-western Patagonia from arid steppe to lakeshores, dense forests, or urban areas.

Cognitive abilities in *V. germanica,* however, have not been studied thoroughly, whereas much research has been done on honeybees with regard to this topic (e.g. Menzel 2009; Cheng 2005; Cheng and Wignall 2006; Giurfa 2007). For instance, honeybees, *Apis mellifera,* can extract visual regularities from their environment and transfer them to novel stimuli (Hateren et al 1990, Stach et al 2004; Giurfa et al 1996). Thus, when presented with novel patterns belonging to a previously learned category (i.e. pattern orientation or symmetry), honeybees choose the appropriate patterns in spite of the novelty of the structural details (Hateren et al. 1990, Stach et al. 2004; Giurfa et al. 1996). Moreover, honeybees have the ability to generalize visual stimuli, as demonstrated in transfer tests (Whener 1971). As an example, they can detect and generalize symmetry or asymmetry (Giurfa et al. 1996) and can transfer information acquired about a previously rewarded pattern to its mirror image or its left—right transformation (Stach and Giurfa 2001). Furthermore, honey bees can extract orientation cues in a pattern from several features, such as individual bars, edges, thin lines, and sinusoidal gratings (Hateren et al 1990); during maze navigation, they can associate a particular stimulus with a particular direction (Zhang et al. 1996; 2000). In addition, when trained with complex patterns that share four edge orientations, honeybees can remember these orientations in their correct positions and generalize their response to novel stimuli which maintain the trained arrangement (Stach et al 2004). The aim our to study whether wasps can discriminate patterns on the basis of their orientation, by evaluating the association between a particular pattern orientation of striped stimuli and the location of a food source. The task required a search of one of four locations for food when presented with a certain orientation and a search at a different location when the stimulus had a different orientation. Moreover, their capacity to generalize this association was analyzed by using new patterns that differed in spatial details, but preserved the learned orientation.

## Materials and Methods

The experiments were conducted in natural, outdoor environments near San Carlos de Bariloche (41° S, 71° W), Argentina, during the period of major activity of *V*. *germanica* wasps (February – April) in 2009. Wasps were trained to feed from a horizontal experimental device consisting of an array (a striped pattern 28 cm diameter) surrounded by four dishes, one of which contained food. When a forager spontaneously arrived at the dish with food, it was marked with a dot of washable paint on the abdomen for further identification. This procedure disturbed wasps only slightly, as they were not captured for marking. Any other wasp visiting the dish was removed in order to work with only one individual per experiment. The wasp collected a piece of meat, then flew away, and returned approximately eight minutes later, on average. Each wasp always approached the array from the same direction each time, as they returned from the nest each time.

**Figure 1.**
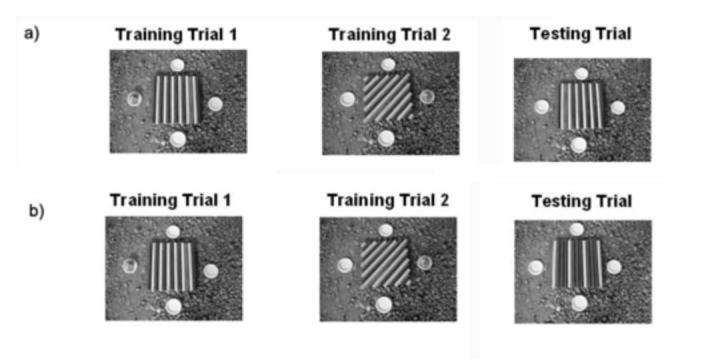
(A) Experiment 1. During training trial 1, *Vespula germanica* first fed from a dish located in a certain position in relation to a striped pattern. In its second visit (training trial 2), the wasp fed from a different location with a different striped pattern. During the testing trial, the striped pattern utilized in training trial 1 was presented. (B) Experiment 2. As in Experiment 1, a certain pattern was paired with one relative position of the feeder. However, in this experiment, training consisted of two trials with a certain stripe pattern paired with a certain food location, and two trials with another stripe pattern paired with the opposite food location. In all cases stripe's width varied in order to present a general pattern, but a different stimulus during each visit. During the testing trial, a stripe pattern with the same orientation as in training trial, 1 but with a different width and color design, was presented. High quality figures are available online

During training trial 1, an individual forager was trained to find food from a white plastic dish (diameter 7 cm) containing 20 g of minced bovine meat. This dish (feeder) was placed either to the left or to the right of a striped array with blue and yellow lines, while three clean dishes were placed at the other cardinal points (Figure 1). The pattern of the striped array had different orientations (Figure 1). We operationally define a reference orientation (N = north, S = south, E = east, W = west) to describe the patterns in terms of the angular difference with respect to this reference. The stimulus types included vertical stripes, i.e. N-S orientation; horizontal stripes, i.e. E-W orientation, and oblique (45°, i.e. NE-SW) relative to the wasps' approach direction. Different patterns were designed: for example, the width of the stripes was varied, while the general pattern (direction of the stripes) was maintained. Also, the visual stimuli was changed, e.g. oblique stripes ascending to the right could be formed by three blue lines and one yellow, two blue and two yellow, or one blue and one yellow, lines varying between 2 and 6 cm in width.

During training, wasps fed from the right or left of this striped array, and paired a particular pattern (i.e. N-S, W-E or NE-SW) with a specific food location (right or left). Side was defined in terms of left and right because the wasps approached from one direction.

### Data collection

During the testing trial, food was removed, and wasp behavior was scored by recording the number of visits made to each clean dish over five minutes. A wasp hovering over a dish or landing on a dish was considered a wasp visit. A hovering episode occurred when the flying wasp remained in the same place, beating its wings for some seconds, over any clean dish without landing on it. Landing, on the other hand, occurred when the wasp touched, with its six legs, any one of the four clean dishes. An observer who sat at approximately 50 cm from the experimental array recorded wasp responses. Each forager was used in only one training-testing sequence. In scoring the data, each location was given 1 point for a hover and 2 points for a landing (Cheng and Wignall 2006; Lozada and D'Adamo 2009). The dependent variable was the score for each location divided by the total score (i.e. visits to all four locations), which is equivalent to the proportion of searching at a certain location.

### Experiment 1

In the first experiment, wasp foragers were trained in two different tasks consecutively (i.e., training trials 1 and 2, respectively), and their response evaluated in the first rewarded situation (i.e., testing trial). This was done in order to determine whether wasps are capable of discriminating between oblique and vertical colored stripe patterns. Wasps were trained to feed consecutively from two different stripe patterns, which were similar in the color and width of stripes and only differed in stripe orientation (as shown in Figure 1). Each pattern was paired with one relative position of the rewarded dish. For example, an individual wasp fed from a certain location to the right or left of a certain orientation pattern. If a certain location was paired with a certain pattern in training trial 1, then during the consecutive training trial 2, both the pattern of the striped array and the location of the feeder were changed. Thus, if a wasp first fed from a dish to the left of the array with vertical stripes, then in the second training trial, food was located to the right of an array with oblique stripes. In this way, for each wasp, food position and stripe pattern orientation differed between training trials, as each training trial consisted of one feeding visit. Thus, each learned pattern required a different wasp response, since food was presented in a diametrically opposite position. After training trial 2, the testing trial began; four clean, empty dishes were placed in the same location as training trial 1. We operationally defined target location as the location of food during the first trial, non-target as the location of food during the second trial, and non-learned as the other two locations, never rewarded. Therefore, if the wasp had learned to associate a certain stripe pattern with a certain food location (i.e., target location) a higher number of visits would occur at that target location. This correct choice would occur even though wasps had learned a different food location, associated with a different stripe pattern (not presented at the testing trial).

### Experiment 2

In the second experiment, a certain pattern was paired with one relative position of the feeder as previously described. However, in this experiment, training consisted of four trials. In two training trials a certain stripe pattern was paired with a certain food location, and in the other two another stripe pattern was paired with the opposite food location. In all cases, the width of the stripe pattern varied in order to present a general pattern, but a different stimulus during each wasp visit. For example, if a wasp first fed from the left of a striped array with vertical stripes, and then, from the right of a array with oblique stripes, subsequent trials would maintain this relative position of the food with respect to the pattern; although the width of the stripes would change (Figure 1). The width of the stripes differed as these could be formed by one yellow stripe and one blue stripe alternately, two yellow stripes and two blue stripes alternately, or three blue stripes and one yellow stripe alternately. These different arrays were randomly presented. After training, during the testing trial, four empty dishes were placed around one of the two stripe patterns previously offered, but with a different width design. In this way, if wasps had learned the relative position of food in relation to the stripe pattern during the testing trial, they would generalize this learned pattern, visiting the correct location more frequently than the others. In the control group, food was presented in a certain location, which was not paired to a specific stripe pattern. For example, if a wasp first fed from the left of a striped array with vertical stripes, and then, from the right of an array with oblique stripes the third trial would consist of presenting food either at the right of an array with vertical stripes, or at the left of an array with oblique stripes. Therefore, wasps could not associate a certain food location to a certain stripe pattern. As in the first experiment, the target location was that paired with the stripe pattern presented at the testing trial. The non-target was that learned during training, but not presented at the testing trial, and the non-learned locations were those never rewarded. For the control group, the same terminology of target, non-target, and non-learned locations was used for comparative purposes.

Statistical comparisons of the frequency of wasp visits to any of the four feeder locations were conducted with Friedman ANOVA. Pairwise comparisons were done using Wilcoxon matched pairs test or the t test. Comparisons of data between experimental and control groups were conducted with the t test. Comparisons of wasp searching at each location were analyzed by Chi Square test.

## Results

### Experiment 1

**Figure 2.**
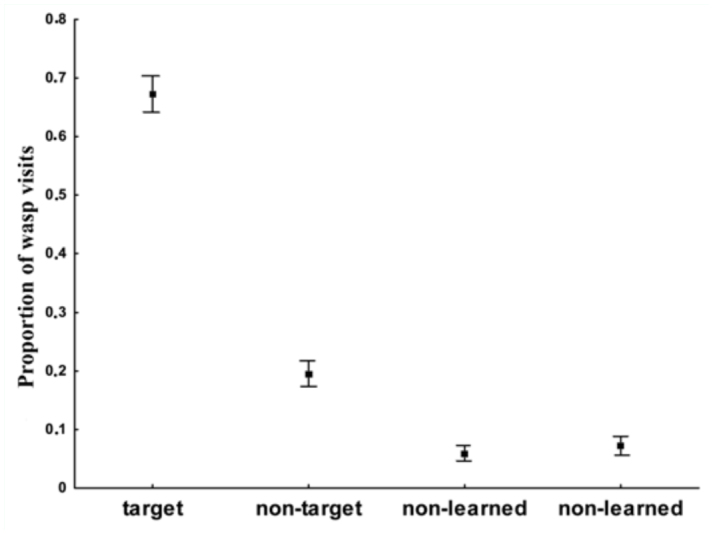
Experiment 1: Proportion of *Vespula germanica* visits to the four dishes in the testing trial (Mean + standard error). Target refers to the food position paired with the pattern used in training trial 1, and non-target refers to the feeder position paired with the pattern used in training trial 2; and non-learned, to the other two dishes, never rewarded (N= 24). High quality figures are available online

Wasps visited the target location significantly more frequently than the non-target location (t = 9.66, df = 21, p < 0.0001) (Figure 2). The proportion of wasp searching at the target location was significantly greater than expected by chance alone (mean = 67%, SE = 3.6, χ^2^ = 923.51, p < 0.0001). The 95% confidence interval about the mean exceeded the chance value of 0.25. Moreover, the proportion of visits to the non-target location was significantly higher than to the non-learned locations (t = 5.36, df = 21, p < 0.0001). The proportion of wasp searching at the non-target location was 19% on average (SE = 2.5), a value significantly below that expected by chance level (χ^2^ = 62.29, p < 0.0001). As expected, non significant differences existed between the proportion of visits to the two non-learned locations (t = 0.64, df = 21, p > 0.53). The average proportion of wasp searching at each of the other two locations was 5.9% (SE = 3.2) and 7.2% (SE = 3.9), values significantly below those expected by chance (χ^2^= 201.54, p < 0.0001; χ^2^ = 171.26, p < 0.0001, respectively) (Figure 2).

### Experiment 2

**Figure 3.**
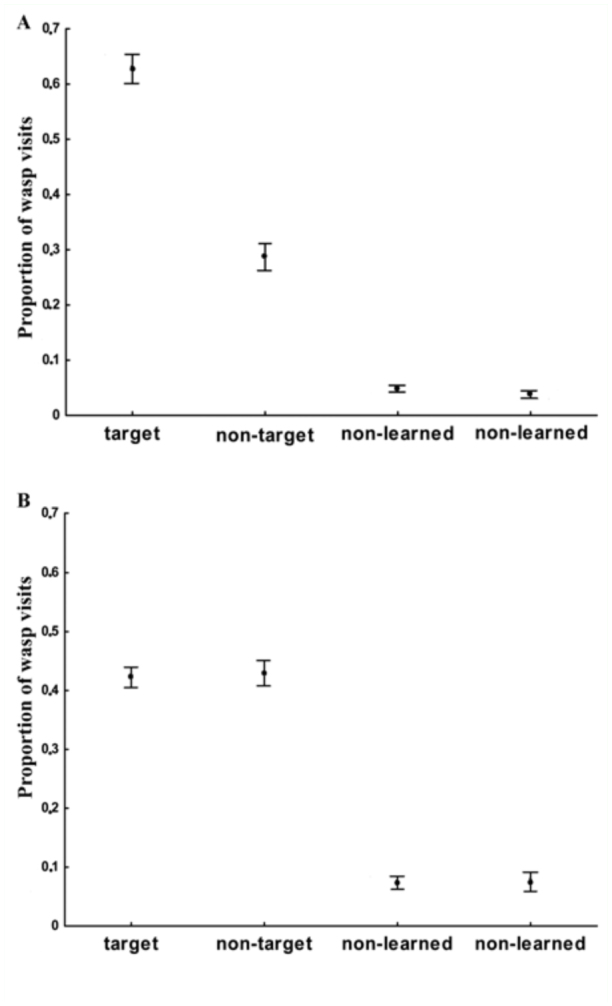
Experiment 2: Proportion of *Vespula germanica* visits to the four dishes during the testing trial (Mean + standard error) for the (a) experimental and (b) control groups. Target refers to the food position paired with the pattern used in training trial 1, non-target refers to the feeder position paired with the pattern used in training trial 2, and non-learned to the other two dishes, never rewarded (N=27). High quality figures are available online

In the experimental group, wasps visited the target location at a significantly higher frequency than the other three locations (χ^2^ = 68.76, N = 27, df = 3, p < 0.00001) (Figure 3). The proportion of searching at the target location was significantly greater than that expected by chance alone (mean = 62%, SE = 3.0; χ^2^ = 785.36, p < 0.0001). The 95% confidence interval about the mean exceeded the chance value of 0.25. Significant differences in wasp searching existed between the target and the non-target locations (Z = 4.13, p < 0.0001). Moreover, the proportion of visits to the non-target location was significantly higher than to the non-learned locations (Z = 4.51, p < 0.0001; Z = 4.46, p < 0.0001, respectively). The proportion of wasp searching at the non-target location was 29% on average (SE = 3.0), a value above chance level (χ^2^ = 82.52, p < 0.0001). As expected, non significant differences existed between the average proportion of visits to the two non-learned locations (Z = 1.90, p > 0.05). The proportion of wasp searching at the other two locations was 5% (SE = 1.0) and 4% (SE = 1.0) both significantly below the value expected by chance (χ^2^= 217.57, p < 0.0001; χ^2^ = 242.79, p < 0.0001, respectively).

In the control group, wasp visits significantly differed among the four locations (χ^2^ = 36.69, N = 15, df = 3, p < 0.00001) (Figure 3). The proportion of wasp searching at the target location was 42% on average (SE = 1.7), and at the non-target location the proportion was 43% on average (SE = 2.1); both values were above chance level (χ^2^ = 107.18, p < 0.0001; χ^2^ = 115.03, p < 0.0001). The proportions of wasp searching at the two non learned locations were 7.3% and 7.5% on average (SE = 1.1 and 1.6, respectively), both were significantly below the values expected by chance (χ^2^= 103.38, p < 0.0001; χ^2^ = 103.13, p < 0.0001, respectively). In contrast to the experimental group, non significant differences existed between the proportion of visits to the target and non-target location (Z = 0.31, N = 15, p > 0.75), whilst the proportion of visits to the target and the two non-learned locations were significantly different (Z = 3.41, N = 15, p < 0.0007; Z = 3.41, N = 15, p < 0.0007), as found when comparing the proportion of visits to the non-target and both non-learned locations (Z = 3.41, N = 15, p < 0.0007; Z = 3.41, N = 15, p < 0.0007). Non significant differences were observed between the two non-learned locations (Z = 0.31, N = 15, p > 0.75).

When comparing the proportion of searching at the target, significant differences were found between the experimental and the control groups (Z = 3.79, p < 0.0002, N_1, 2_ = 22, 15) (Figure 3). The proportion of searching at the target location was 62% on average (SE = 3.0), while in the control group it was 42% on average (SE = 1.7). Furthermore, significant differences were observed between the experimental and the control groups in the proportion of visits to the non-target (Z = -3.40, p < 0.0007, N_1, 2_ = 22, 15). The proportion of wasp searching at the non-target location was 29% on average (SE = 3.0) for the experimental group and 43% on average (SE = 2.1) for the control group.

## Discussion

The present study shows that *V. germanica* wasps are able to discriminate between two striped patterns with different orientation and to generalize their choice to a new pattern that differed in quality, but presented the same orientation. They searched most at the appropriate target location, far less frequently at the location the other stimulus orientation, and hardly at all at the unrewarded locations.

*Vespula germanica* wasps showed a great ability to discriminate between oblique versus vertical patterns, associating a certain orientation pattern with a particular food location. Previous experiments have shown similar sensory motor learning capacities in this species (D'Adamo and Lozada 2009), as they associated specific colored protruding landmarks with a certain motor response. Thus, if wasps associated a blue landmark with food placed to the west of the landmark, and a yellow landmark with food to the east, then this learned motor response prevailed over the last rewarded location. Instead of protruding landmarks, the present study used horizontal arrays, which included complex, distinct orientation patterns instead of different colors. Although greater complexity was introduced into the stimulus configuration, wasps were able to learn discriminative tasks with only one trial. The use of four locations in the experimental design allowed us to distinguish between the appropriate rewarded locations vs. the other rewarded one and those never rewarded. The results showed rapid learning and a lack of task interference from learning two different task requirements. This differs from the interference effects found in honeybees (Cheng and Wignall 2006). In their study, bees encountered much *response competition* (i.e. an ambiguity that can produce competition between the response appropriate for task 1, and that appropriate for task 2, which leads to worse test performance). In contrast, in the present study wasps performed in the tasks effectively. This could be due to the fact that the orientation differentiation (required in this study) might be easier than color discrimination (required in Cheng and Wignall's study). Moreover, in the present study both the stripe orientation and the shape of the stimulus differed since from the approach direction one is square while the other is a rhombus. Thus, a shape cue plus an orientation cue combine to provide more spatial cues than a mere color difference. Another explanation could be that the multiple training trials carried out by Cheng and Wignall could have induced task interference of the second task on the first task. In this sense, more training on task 2 in their experiment might have created the response competition. However, as species and experimental protocols differed between studies, firm conclusions about the source of these differences cannot be established.

Results from our study suggest that *V. germanica* wasps could be positively transferring a learned orientation pattern despite variations in a new stimulus. When presented with a novel layout, which belonged to a previously learned category, wasps chose the appropriate pattern despite the novelty of the structural details. This capacity to generalize visual stimuli has been thoroughly demonstrated in the traditional model of honeybees (Wehner 1971; Giurfa et al. 1996; Stach and Giurfa 2001; Hateren et al 1990; Ghirlanda and Enquist 2003), but to our knowledge, this is the first study suggesting this capacity in a social wasp. Positive transfer learning has been studied in *Apis mellifera* by training bees with much more complex setups than employed in this study (Giurfa 2007). In the bee studies, stimuli consisted of a series of complex patterns sharing a common layout comprising four quadrant orientations. The bees learned these orientations simultaneously in their appropriate positions and transferred the associated response to novel stimuli that conserved the trained layout (Stach et al. 2004). In this foraging paradigm, bees can deal with stimuli they have never experienced previously and therefore do not predict food presence. Cognitive plasticity could be highly beneficial for an invasive species, which is continuously dealing with novelty and uncertainty. Although the present study was carried out with a much simpler experimental design than those conducted in honeybees, the preliminary results suggest that *V. germanica* is capable of generalizing visual patterns which differ in quality, but present the same orientation.

This learning capacity supports previous studies showing the existence of diverse flexible mechanisms in this species while foraging, integrating old and new experiences after very few learning episodes (e.g. D'Adamo and Lozada 2009; Lozada and D'Adamo 2009). Wasps exploiting a rich food source that suddenly disappears continue visiting the site for a period of time, which is related to the number of feeding visits wasps had previously experienced (Lozada and D'Adamo 2006). Moreover, the colored array most recently associated with food was prioritized over a formerly learned colored array, integrating the saliency of the last learned cue and iterative rewarded experiences (Lozada and D'Adamo 2009). In addition, it has been shown that one-trial learning is sufficient for this species to establish an association between diverse cues and food reward in different contexts (D'Adamo and Lozada 2009; Lozada and D'Adamo 2009).

Studying whether or not similar capabilities are shared by other social insects, such as *V. germanica*, could be interesting. We would like to highlight the relevance of studying choices made by free flying wasps in different natural circumstances. In our experimental design, meat was removed after wasps had fed on it and their behavior was studied while relocating a food source. Decision-making requires reference to both current and remote information in the context of the animal's requirements; therefore studying choices made by wasps in natural conditions may be useful when evaluating decision-making. This study presents supplementary evidence of the cognitive plasticity of this invasive social wasp, which, we hypothesize, could be related to its invasive success.
